# Hope amidst crisis: exploring perinatal mental health and family dynamics in out-of-home care through virtual assessments during the UK COVID-19 response

**DOI:** 10.3389/fgwh.2024.1343944

**Published:** 2024-02-12

**Authors:** Udita Iyengar, Jessica Heller-Bhatt

**Affiliations:** ^1^ Department of Psychosis Studies, Institute of Psychiatry, Psychology, and Neuroscience (IoPPN), King’s College London, London, United Kingdom; ^2^ Attachment Based Training, Denmark, WA, Australia; ^3^ The Harvest Clinic, Kin Kin, QLD, Australia

**Keywords:** parenting, attachment, perinatal mental health, COVID-19, virtual dyadic assessments

## Abstract

Caring for a young child exposed to early trauma, along with caregiving stress and heightened by the impact of lockdowns as a result of the COVID-19 response, may compromise the development of the parent-child relationship. Understanding a foster carer's attachment history and considering relational dynamics through an attachment lens may shed light on areas they need support in, to enhance their parenting capacity for vulnerable children. The feasibility of collecting and coding observational data and attachment interviews of foster carers and their children, when conducted remotely during COVID-19, needs to be explored. This perspective piece considers the impact on infant and perinatal health in the context of COVID-19 with particular emphasis on relational dynamics and attachment assessments, using a case study of a foster carer and her child in an out-of-home-care placement. Understanding these dynamics is crucial for safeguarding the well-being of both caregivers and vulnerable children during this challenging time.

## 1 Introduction

The period of pregnancy and the initial 12 months postpartum, referred to as the perinatal period (1), is a particularly critical time marked by significant biological, psychological, and physiological changes for both mother and child (2–5). Adverse experiences during the perinatal period can have a profound impact on children, sometimes leading to a young child experiencing maltreatment in early life resulting in the removal from their home to improve safety and well-being. Children who are removed from their home due to maltreatment are at increased risk of a range of adverse mental and physical health outcomes, including self-harm, suicide attempts, and suicide (6–8). However, such negative effects can be reversed if placed in nurturing out-of-home-care^1^ early in life (9), and foster carers can have positive benefits for a child's emotional and psychosocial development (10). Over the last few decades, research has focused on developing nuanced assessment and intervention approaches to address the often complex needs of infants within their primary caregiving system (11–15). Studies examining infant mental wellbeing and early development have provided robust support for the association between maternal sensitivity and child self-protective attachment strategies, drawing attention to the significance of early interventions with struggling mothers (16) with families with a history of abuse or neglect. The Best Services Trial (BeST?), for example, is a UK Randomised Control Trial which seeks to evaluate the efficacy of an infant mental health-based relational assessment and intervention model (17, 18), which has an approach to both assessment and treatment in cases of maltreatment for looked after children in out-of-home care.

## 2 The global impact on infant and perinatal health in the context of COVID-19

In times of health-related disasters, the support- and lack thereof- to parents becomes a salient topic in the prevention and amelioration of perinatal mental health problems. Perhaps this applies even more so to the out-of-home care population, dominated by children with high needs who are likely to be at increased risk of suffering adverse impacts as a result (19).

Some of the surrounding circumstances around the outbreak of the coronavirus disease (COVID-19) had a profoundly negative impact on mental health in the general population, and young children in the out-of-home-care population were particularly vulnerable to the impact of the COVID-19 response in terms of mental health and development. Stay-at-home mandates and lockdowns along with the uncertainty surrounding COVID-19 likely exacerbated an already stressful experience of being removed from one's parents. In Australia, an investigation into the effects of lockdowns revealed an associated increase in mental health issues, most notably for women with children in coupled households and between the age of 20 and 29 (20). Quarantine procedures were also seen to be accompanied by post-traumatic stress symptoms in individuals concerned (21). A systematic review of perinatal mental health experienced during the COVID-19 outbreak suggests risk and protective factors experienced by mothers in the perinatal period (2). Risk factors included previous mental health struggles and financial stress, while protective factors pertained to accessing information and knowledge about the impact of COVID-19 on health, increased perceived social and marital support, as well as greater sleep and physical activity. Lockdown restrictions also precluded many birth families from having face-to-face contact with their children, which led to a rapid shift to virtual court hearings, remote parenting capacity assessments, and video/audio-based family contact sessions. We wondered then, how feasible would the use of virtual assessments be when trying to capture aspects of relational dynamics between carer and young children in out-of-home placements?

### 2.1 How can we assess dyadic interaction in the context of self-protective attachment behaviours?

Attachment theory is a particularly useful framework in understanding how formative relationships shape our psychological organisation and behaviour across the lifespan and constitutes an array of self-protective strategies organised around danger (22–25). In infants and younger children, attachment is a relationship-specific characteristic rather than a person-specific factor. This is to do with the developmentally expected dependence on their caregivers, which lessens with ongoing neurological, emotional, and social maturation. While children solely depend on their caregivers for comfort and protection in early childhood, their use of self-protective behaviours is organised around their primary attachment figures to maximise their chance of having these needs met (26).

Considering the significant, adverse perinatal impact of social isolation in the context of the COVID-19 response, we were interested in whether certain psychological assessment tools could capture clinically important aspects of the parent-child relationship utilising online facilities, specifically, the Adult Attachment Interview (27, 28) and the Toddler Care Index (29). The AAI considers childhood experiences and how they might affect thoughts and behaviour in the present. It is audio/video-recorded to enable transcription for evaluation purposes and has been employed in a range of studies investigating child development as a function of primary caregiving experiences (30–33). The TCI is an adapted version of the dyadic-specific, well-validated parent/child assessment, the Infant CARE-Index (29). Its screening ability for dyadic risks for children and their families make it unique in addition to being an inexpensive, easy-to-conduct 5 min procedure using a simple play interaction. The TCI yields scores regarding caregiver sensitivity, control, and unresponsiveness, and about the toddler's levels of cooperativeness, compulsivity, and passivity. It generates information regarding the level of dyadic synchrony in the context of probable risk to the infant's future development (29, 34).

As both the AAI and the TCI are audio/video recorded, they appear especially suitable for being employed digitally. Using online recording features enables clinicians to audio record the AAI while automatically producing a written transcript for further analysis. Similarly, the TCI can be done simultaneously by setting up play in front of a digital device, with the clinicians recording the procedure in real time using virtual platforms. Alternatively, one of the caregivers facilitates the recording by placing a phone or tablet overlooking the play interaction. For the latter, brief instructions to the caregivers regarding the procedure and the recording set up are required.

### 2.2 Supporting foster carer-child dyads using attachment-based assessments: a case study

The following is a summary of a case study of a foster carer and her foster child, an 18-month-old boy in the foster care system in the UK. The foster carer agreed to participate in a virtual study to understand Attachment Patterns of Foster Carers and their Children in Care during the COVID-19 outbreak. The study utilised an online AAI and TCI video recording of carer and child interactions completed by and sent in via the foster carer. Both AAI and TCI were analysed and advised by trained coders. The foster carer gave informed consent and ethical approval was granted by King's College London Research and Ethics board (HR-19/20-17203).

#### 2.2.1 Case history

The foster child had been in the care of the foster mother for 4 months when the assessments were conducted, following one year after the UK government-mandated lockdown in March 2020. The foster child had previously resided in several placements with family members for most of lockdown before joining his current out-of-home care arrangement, which also included two biological children living at home. The foster carer described him as “high energy, very enthusiastic, affectionate, and engaged”. Before arriving at his placement, the child was said to have “little to no routine”, and “the first week he would have awful night terrors, had little ability to follow instructions, and was very unpredictable”. The foster carer reflected that “I suspect that all of his chaos behaviour is linked to not being safe and is triggered by his contact visits with his mother”. However, after 4 months in her care, the foster carer added that “he is growing with the idea that you can have permanent adults in your life… I'm no expert in attachment, but people would say, he is really attached- he attaches to many adults, rather than have a sense of danger, he will terrifyingly talk to strangers”. Whilst, on the surface, it may appear that the child was not concerned about danger, his indiscriminate approach of and rapid rapport building with strangers can represent important self-protective strategies. Functionally, this behaviour can be understood as attempting to build relationships with various adults who may provide a level of safety when the child cannot access a predictably and consistently comforting and protective primary caregiver.

When asked what she wished for her foster child in the future, the foster carer stated “I want him to thrive- he has so much potential. He is so smart, he is so switched on, especially after his early start—with investment and guidance he could really thrive at life and be a successful adult.”

#### 2.2.2 Assessment findings

The foster carer's behaviour came across as engaging, pleasant, caring, and compliant, and she had a seemingly spontaneous and natural interaction with her foster child, one that was brimming with affection, patience, and turn-taking. The AAI yielded information regarding the foster carer's ability to use a level of reflective thinking that is important in trying to attune to her foster child's needs. Her interest in learning more about the process of attachment, including effective strategies to support her foster child as best as possible, was also evident.

Discourse analysis of the AAI transcript offered insight into the foster carer's tendency towards compulsive caregiving of others, performing to a high standard, complying with others in perceived authority (including the interviewer), and behaving in a conflict-avoidant manner (Type-A attachment strategy). The emotionally inhibited Type-A strategy can vary from mild to extreme and, based on the case study's AAI findings, the foster carer's use of self-protective Type A behaviours was estimated to be mild and outside of the clinical range. There were no indicators of unresolved trauma, loss, or markers for depression, nor were there signs placing the foster carer into a risk category. Generally, such self-protective strategies are associated with individuals’ early life experiences of having to inhibit their negative internal states, thoughts, and opinions at the cost of forfeiting more transparent communication with others. In these cases, safety tends to be associated with remaining distant from others rather than with seeking comfort and protection through co-regulation and interpersonal closeness.

Findings obtained from the TCI suggested that the child used predominantly normative “Type C” strategies. Specifically, the child's behaviours ranged from threatening to becoming upset to acting innocent and confused. Intermittent aspects of aggressiveness and “feigned helplessness” were also observed, the latter representing behaviour that functions to elicit parental caregiving. Children using a Type C strategy often behave in unpredictable ways, while demanding predictability from their caregivers (28). They tend to split negative affect between vulnerable (being desirous or fearful of comfort) and invulnerable internal states (being angry). With this strategy, children tend to oscillate between these two states, exaggerating one over the other, while simultaneously inhibiting the opposite state. This behaviour is used contingently in response to the parent to increase parental predictability and consistency. Gauging the level of dyadic synchronicity between carer and child indicated a relatively low dyadic synchronicity and within the range where interpersonal problems may be present. For such dyads, a supportive therapeutic intervention may be needed to prevent interpersonal problems from exacerbating and a possible placement future placement break down. Children who have previously experienced relational trauma may display behaviours which impact on the quality of caregiving (35), even for sensitively attuned, reflective, and experienced caregivers.

#### 2.2.3 Reflections on case formulation and intervention planning

Given the degree of vulnerability of children placed in out-of-home care, comprehensive assessments involving dyadic dynamics seem indispensable to case formulation and planning of therapeutic support. In our case study, understanding the child's use of mild to concerning Type C strategies with his foster carer alerted us to his need for increased predictability in his every-day routines, consistently attuned caregiver responses, gentle yet firm boundary setting, and emotional connection within his immediate caregiving system. Sharing his placement and his caregiver attention with biological children may be challenging for him and increase his tendency to use attention-eliciting behaviours to ensure he receives the needed emotional connection. Meanwhile, in view of his foster parent's tendency towards conflict-avoidance and her own developmental experiences of being compliant with others, we may consider how parenting a child with threating and conflict-enhancing behaviours could potentially become a parenting challenge. However, the reflective statements the foster carer had about her child in care and herself could be considered protective factors that benefit her child's development.

A synthesis of the case study's data revealed the importance of collating relational information and the interpersonal function assigned to the observed behaviour in devising appropriate therapeutic support services for children in out-of-home care. For many foster carers, children are perceived as doing better when they obey and fit in, and so making use of carer's observed reflective capacity seems important to pre-empt difficulties from arising in the relationship. The fine nuances made available through assessments of attachment behaviours, such as the AAI and TCI, suggest invaluable sources of information regarding interpersonal functioning within the family system.

## 3 Discussion

Since adapting to COVID-19 and utilising remote assessments, emerging research regarding digital mental health services has shown preliminary support for the efficacy and feasibility of online services (36, 37), for a recent meta-analysis). Therefore, we posed the question: can virtual attachment assessments be employed going forward, given the use of telehealth that has remained even past the pandemic, and are there things to be learned from this case study? We believe they can and, whilst face-to-face contact will likely remain the gold standard for the most effective, ethical, and meaningful way of working with vulnerable individuals, both the AAI and the TCI naturally lend themselves to electronic delivery.

### 3.1 Strengths and limitations and personal reflections

This pilot study's primary strength was that it demonstrated the ability to adapt an in-person observational attachment-related assessment to a virtual format, despite the complexities and circumstances surrounding the COVID-19 outbreak. Particularly, as this was a time when many research studies were being halted due to stay-at-home mandates, the option to continue with a virtual research study focused on parent-child interactions was advantageous. Other studies have highlighted the benefit for attachment-based assessments [such as Attachment and Biobehavioral Catch-up (ABC)] to be delivered virtually during the COVID-19 outbreak, suggesting improved parental sensitivity when delivered through a telehealth or hybrid format (38, 39). Also pointed out the need for the clinical adaptations and maintaining safety when delivering attachment-based therapy in a virtual space. However, utilizing the DMM framework, specifically the TCI and AAI, enabled a unique combination of virtual clinical assessment of foster parents and their children in care, which, to our knowledge, has not been explored prior to this perspective.

The use of video technology in the delivery of the TCI and AAI to foster carers and their children lends itself as a flexible, inclusive, and innovative evaluation method, and, for some families, may break down geographical barriers and facilitate a more accessible learning environment.

There were several limitations of this study, however, mainly the small sample size. The outbreak of COVID and enforced lockdowns led to challenges with recruiting participants in a virtual attachment-based study in the social care and fostering system. Social workers and foster carers were faced with enormous pressure to adapt their work and caring tasks (such as meetings and appointments) to a virtual space, and recruitment into an optional research study was not a high priority for many families, therefore, only one subject was enrolled in the study.

The use of video technology was also a limitation. Using video technology has shown to alter the interview dynamic by being in a home setting (40), and our case study included numerous interruptions and distractions which interfered with the coding of both the AAI and TCI due to the non-standard assessment. Drawbacks to employing the TCI through instructing caregivers for filming are the chances of the interaction not yielding a full view of the child, limited view of the caregiver, muffled audio, and the child becoming distracted by the filming device. However, it is important to note that the procedure can be repeated, if necessary, without detriment to the findings’ validity.

Lastly, the home setting had a larger impact when administering the AAI, as both the researcher and participant had young children at home due to school closures, which caused multiple distractions when conducting the interview and coding the transcript. Upon further reflection, a few specific things may have inadvertently forged an unintended connection between the researcher and the participant: observing each other's living spaces, unexpectedly encountering family members on camera, and in the context of forced lockdown and experiencing social isolation, engaging in conversation exploring early life and perinatal as well as parenting experiences between the researcher (who herself was in the perinatal period). While therapeutic rapport building during the AAI interview is important, fostering a therapeutic bond is not meant to occur during research, and future studies should be mindful of this.

### 3.2 Future directions and looking forward

Parental capacity to accurately perceive and interpret the child’s signals is seen to be an essential aspect in the development of children's social and cognitive pathways (41). We argue that information regarding parents’ responses towards their children's bid to communicate these needs are of particular diagnostic value and can be obtained through assessments that focus on the type and quality of the dyadic mother-child relationship (12), especially for a vulnerable population, such as children in out-of-home placements. The enduring commitment of foster carers is to provide a positive environment for their children in-care and ideally improve child outcomes, and we believe that any attempt to ameliorate the effects of perinatal trauma is paramount to a child's future success.

## Data Availability

The datasets presented in this article are not readily available because the dataset contains audio and video and written transcripts that contain personal and identifiable information, which participants did not give consent to share and will not be shared. Requests to access the datasets should be directed to UI.

## Footnotes

^1^We refer to out-of-home-care as including residential facilities, foster care, group homes, or other arrangements where a child or individual is placed in the care of someone other than their biological family.
